# Nutrient supply from marine small-scale fisheries

**DOI:** 10.1038/s41598-023-37338-z

**Published:** 2023-07-13

**Authors:** Daniel F. Viana, Jessica Zamborain-Mason, Steven D. Gaines, Josef Schmidhuber, Christopher D. Golden

**Affiliations:** 1 Department of Nutrition, Harvard T.H. Chan School of Public Health, Boston, MA 02115 USA; 2 Ocean Conservation, World Wildlife Fund, Washington, DC 20037 USA; 3 Bren School of Environmental Science and Management, University of California, Santa Barbara, Santa Barbara, CA 93106 USA; 4 Markets and Trade Division, Food and Agriculture Organization, Rome, Italy

**Keywords:** Ocean sciences, Risk factors

## Abstract

Over 2 billion people are unable to access safe, nutritious and sufficient food year-round. While global fisheries are considered key in providing essential nutrients to hundreds of millions of people around the globe, the specific contribution of small-scale fisheries to the nutrient supply given other available food supplies is unknown. Here, we combined multiple global databases to quantify the importance of marine small-scale fisheries to national-level nutrient supply of coastal populations. We found that, on average across assessed nutrients (iron, zinc, calcium, DHA + EPA and vitamins A and B_12_), small-scale fisheries contributed about 32% of overall global seafood nutrient supply, 17% of the nutrient supply from animal-sourced foods and 10% of nutrient supply from all foods. These global averages, however, underrepresent some key roles of ocean-based foods. Combining nutrient supply estimates with global estimates of inadequate nutrient intake, we found that about half of coastal countries that have a mean inadequate intake of at least 50% across assessed nutrients (iron, zinc, calcium, DHA + EPA and vitamins A and B_12_) rely on small scale fisheries for at least 15% of mean nutrient supply, and many rely on small scale fisheries for more than 30% of mean nutrient supply. Catch from small-scale fisheries is particularly important for the supply of vitamin B_12_, calcium and DHA + EPA, representing up to 100% of supply in selected countries. Our study demonstrates the significance of small-scale fisheries for nutritionally vulnerable coastal populations, emphasizing how effective fisheries management can contribute to public health.

## Introduction

Unhealthy diets and insufficient food are the leading cause of death globally^1^, with over 2 billion people deemed food-insecure^2^. Concurrently, more than 3.3 billion people around the world depend on fish for at least 20% of their animal-protein intake, and in many developing countries, seafood accounts for more than 50% of the total animal-protein intake^3^. In addition to protein, seafood provides critical contributions of nutrients such as iron, zinc, calcium, iodine, selenium, vitamin B_12_, and fatty acids^4,5,6^. The human body needs consistent access to small quantities of these micronutrients to enable proper physiological and immune function^7^. Beyond their role in filling micronutrient gaps in nutritionally vulnerable communities, seafood can displace the consumption of less healthy meats^4^, and two servings per week can significantly reduce the risk of certain non-communicable diseases^7^ and prenatal and child mortality^8^, thus increasing life expectancy and quality^9^.

Small-scale fisheries (SSF) can be broadly defined as fishing activities occurring on smaller boats or on foot, for commercial and/or subsistence purposes^10^, yet there is still no consensus among countries on where to draw the line between small-scale and industrial fisheries^11^. Here, we consider catch from small-scale fisheries as the catch from artisanal and subsistence sectors defined by the Sea Around Us project, which is the data used in the analysis^12^. Catch from the small-scale fisheries sector can contribute to human nutrition through two main pathways: direct seafood consumption and fisheries-derived income that is redirected toward purchases that improve nutrition. Seafood sold within communities or at local markets can be more affordable than other animal proteins such as beef or chicken^13^, providing an important nutrient source for coastal communities that do not have access to broader food markets. Seafood sales are also an important source of income for fishers and other actors along the supply chain, which can have positive effects on a household’s food purchasing power^14^. In addition, SSF catch provide a food source with low carbon and environmental footprint compared to other terrestrial animal sourced foods, thus contributing to the sustainability of coastal communities and the planet^15^.

**Figure 1:**
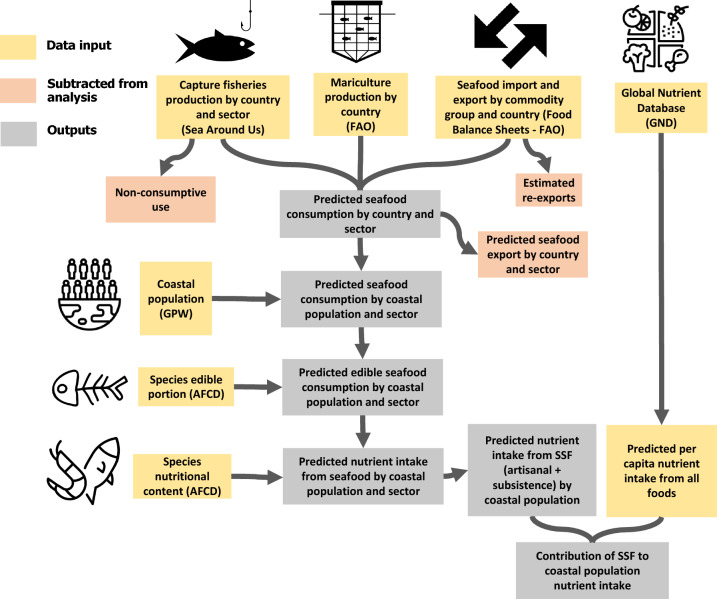
Conceptual diagram of the analysis components and integrated data sources.

Despite increased global attention to the potential role of fisheries in food security and human nutrition^4,5^, no study has estimated the contribution of small-scale fisheries to nutrient supply on a global scale. Past global studies have focused on the importance of capture fisheries and aquaculture to human nutrition^4,5^ but haven’t focused specifically on small-scale fisheries. Global studies that focused on small-scale fisheries highlighted their importance for food security^16^ but did not focus on nutrient supply for coastal communities. To address this knowledge gap, we combined information on global catch^17^, aquaculture production^18^, seafood trade^18^, nutrient composition of aquatic species (AFCD)^4^ and global nutrient supply (GND)^19^ and assessed the relative importance of marine small-scale fisheries to the supply of zinc, iron, protein, vitamin B_12_, omega-3 long-chain polyunsaturated fatty acids docosahexaenoic acid (DHA) and eicosapentaenoic acid (EPA) (hereafter referred as DHA + EPA) and calcium (Fig. 1). Specifically, we analyzed the relative contributions of marine SSF to nutrient supply at national levels relative to: (1) all other seafood producing sectors, (2) all other animal-sourced foods, and (3) all other foods. Next, we calculated the contribution of SSF to national nutrient supplies in relation to estimates of inadequate nutrient intakes, allowing for classification of importance and vulnerability to shocks in SSF production.

## Results

### The importance of SSF as a part of animal-sourced foods

**Figure 2:**
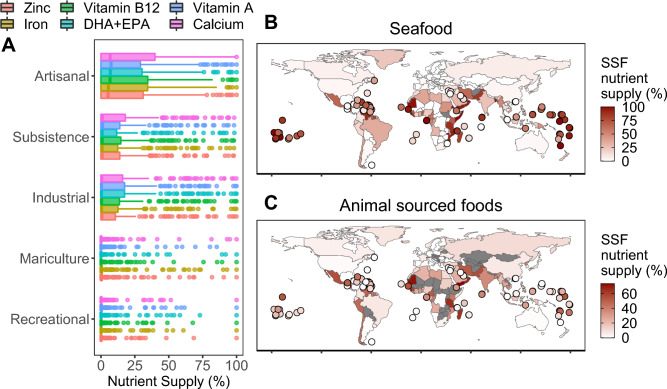
Contribution of small-scale fisheries (SSF) to nutrient supply relative to (**A**) total nutrient supply from all seafood producing sectors (additionally including industrial and recreational fisheries, and mariculture), where each point represents a country; (**B**) average nutrient supply from all seafood producing sectors; and (**C**) average nutrient supply from all animal-sourced foods (fish, beef, veal, dairy, pork, poultry and sheep). Nutrient supply in (**B, C**) are represented as the mean supply across iron, calcium, zinc, DHA + EPA, and vitamins A and B_12_. Countries smaller than 25,000 km^2^ are illustrated as points.

Based on estimates of apparent seafood consumption per country in 2017 (most recent year with complete data), we estimated the relative contribution each seafood producing sector to total national aquatic food nutrient supply (Fig. 2A). On average, small-scale (artisanal and subsistence) and industrial fisheries had the highest relative contribution to nutrient supply followed by mariculture and recreational fisheries. Even though imported seafood was produced by both aquaculture and capture fisheries, we treated it separately because of data limitations (see methods). Therefore, nutrient supply by sector are estimates of the seafood produced and retained within each country and does not account for imported seafood from mariculture or capture fisheries. Consequently, national contribution of the different sectors can be underestimated in countries that import large quantities of seafood.

Next, we estimated the nutrient supply of small-scale fisheries relative to (1) all marine seafood producing sectors and (2) all animal-sourced foods (fish, beef, veal, pork, dairy, poultry and sheep) based on the Global Nutrient Database^19^ (see supplementary Figures S4, S5 and S6 for additional results on all other seafood sectors). On average, small-scale fisheries contributed to 32% of the overall seafood nutrient supply (marine recreational and industrial fisheries and mariculture) across all assessed nutrients (iron, zinc, calcium, DHA + EPA and vitamins A and B_12_). There was large variation across regions and countries (Fig. 2B). Regions such as Polynesia (78%), Micronesia (76%), Caribbean (55%) and Western Africa (50%) have the highest average contribution of SSF to overall seafood nutrient supply. Within these regions, countries such as Sierra Leone (99%), Somalia (99%), Anguilla (98%), Bangladesh (97%), Fiji (96%), Guinea-Bissau (95%) and Mozambique (93%) had the highest contribution of SSF to seafood nutrient supply (see Figure S2 for all nutrients).

Relative to all animal-sourced foods, we found that small-scale fisheries contribute to an average of 17% of the nutrient supply globally. More specifically, SSF contribute to 33% (0–98%) of calcium, 29% (0–96%) of vitamin B_12_, 30% (0–100%) of DHA + EPA, 18% (0–89%) of iron, 14% (0–84%) of zinc and 8% (0–75%) of vitamin A relative to other animal-sourced foods (Fig. 2C). Countries such as Mauritania (73%), Sierra Leone (69%), Oman (66%), Madagascar (65%), and Tanzania (64%) have the highest average contributions of SSF to nutrient supply across assessed nutrients relative to all animal-sourced foods (see Figure S3 for all nutrients).

### SSF are important to dietary nutrient supply

**Figure 3:**
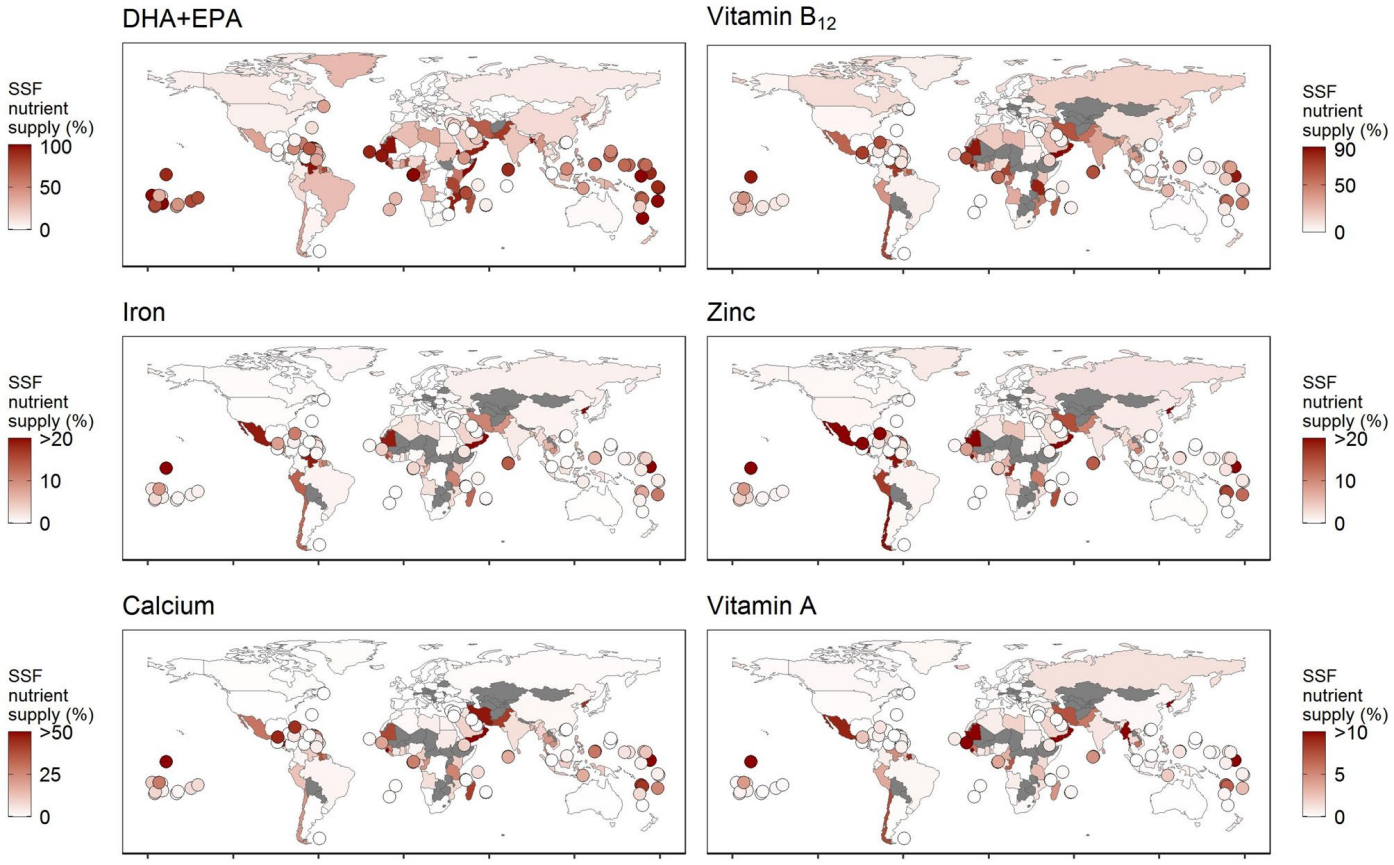
Contribution of small-scale fisheries’ (SSF) nutrient supply relative to the overall nutrient supply from all foods (e.g., grains, fruits, dairy, other animal foods). Countries smaller than 25,000 km^2^ are illustrated as points.

Next, we compared national-level estimates of nutrient supply from SSF to the overall average nutrient supply of all foods consumed within each country (e.g., grains, fruits, dairy, other animal foods), based on the Global Nutrient Database^19^. On average, we found that SSF contribute to about 10% of the total nutrient supply of coastal populations. More specifically, SSF contribute, on average including all countries, to approximately 30% (0–100%) of DHA + EPA, 20% (0–90%) of vitamin B_12_, 9% (0–80%) of calcium, 5% (0–59%) of zinc, 3% (0–44%) of iron, and 2% (0–33%) of vitamin A to overall nutrient supplies (Fig. 3). Yet, these averages belie the local nutritional importance of SSF in some national contexts. For example, for vitamin B_12_, SSF contribute 90% of the supply in Yemen and 89% of the supply in Sierra Leone; for calcium, SSF contribute 79% of the supply in Oman and 52% in Guatemala; for zinc, SSF contribute 47% in Mexico and 32% in Guinea-Bissau; for iron, SSF contribute 25% in Kiribati and 20% in Venezuela; for vitamin A, SSF contribute 20% in Mauritania and 13% in Gambia.

### Nutritionally vulnerable nations depend on SSF micronutrient supply

**Figure 4:**
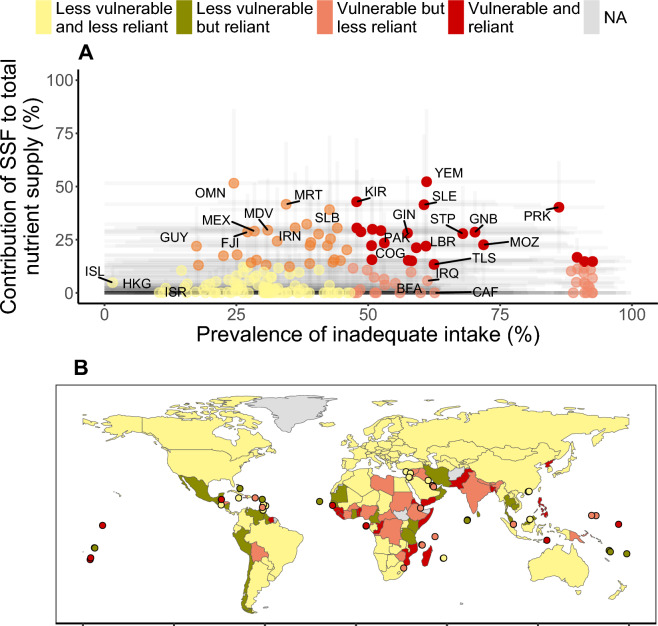
Importance of small-scale fisheries to global nutrition, where (**A**) mean contribution of small-scale fisheries (SSF) to overall nutrient supply across nutrients that are abundant in aquatic species and are important for human health (iron, calcium, zinc, protein, DHA + EPA and vitamins A and B_12_) relative to mean prevalence of inadequate micronutrient intake across all assessed nutrients (% of population—Golden et al. 2021); and (**B**) highlights countries that have high prevalence of inadequate micronutrient intake (vulnerable) and that rely on SSF for the supply of key nutrients (reliant). Countries considered nutritionally vulnerable are those within the 70th and 100th percentile of prevalence of inadequate micronutrient intake. Countries below the 70th percentile are considered less vulnerable to nutritional deficiencies. Countries reliant on SSF are those within the 70th and 100th percentile of overall dietary nutrient supply by small-scale fisheries. Gray areas are those without available data. Three-letter abbreviations represent selected alpha-3 ISO country codes.

Our results show that about half of nutritionally vulnerable coastal countries are also reliant on small-scale fisheries for at least 15% of nutrient supply (Fig. 4). Nutritional vulnerability was derived from inadequate intake calculations^4^ and should be interpreted as the percent risk of nutritional inadequacies within each country. Tropical developing countries are particularly dependent on small-scale fisheries for meeting their nutritional requirements. For example, about one third of coastal nations in Africa have particularly high prevalence of nutrient deficiencies (> 50% of mean inadequate nutrient intake) and have relatively high contributions of marine SSF catch for nutrient supply (> 15% of overall nutrient supply). In countries such as Somalia, Sierra Leone and Madagascar average inadequate intake across assessed nutrients is estimated at about 90%, 60% and 51% respectively and SSF contribute to about 20%, 41% and 30% respectively of overall nutrient supply for coastal populations. In addition, over 60% of pacific island nations are reliant on SSF for nutrient supply (> 15% of overall nutrient supply), but 62% of these nations do not have information on inadequate intake levels^4^. Other countries such as Mexico and Chile have large contributions of small-scale fisheries for nutrient supply but are not vulnerable to insufficient nutrient supplies on a national scale (despite being potentially important in coastal fishing communities). It is important to note that inland countries are not highlighted here but can also have high levels of inadequate intake and be reliant on freshwater fisheries or imported seafood for nutrient supply (see Figure S5 for global patterns of reliance on marine imports).

## Discussion

Our global analysis shows that small-scale fisheries are an important source of key nutrients for vulnerable coastal populations. We estimated that about one quarter of coastal nations around the world rely on small-scale fisheries for more than 15% of mean nutrient intake across assessed nutrients (iron, zinc, calcium, DHA + EPA and vitamins A and B_12_). When considering nutritionally vulnerable countries (defined as a mean national prevalence of inadequate intake over 50%), about half of coastal nations rely on SSF catch for at least 15% of nutrient supply. However, more than 20 nations across Africa, Pacific, Caribbean, and Asia, obtain more than 30% of their nutrient supply from small-scale fisheries catch. In such countries, any decline in availability and/or access to seafood can have significant negative consequences to health and nutrition of the population.

Small-scale fisheries catch is particularly important in populations that do not have access to diverse and rich diets. In many coastal areas around the globe, communities have access to staple foods (such as rice, wheat, corn, cassava) and aquatic foods serve as the sole form of animal-sourced food^20^. For example, a study in Bangladesh showed that fish intake corresponded to about 40% of the recommended Vitamin A consumption and 32% of the calcium consumption^21^. And, household surveys in the Pacific islands demonstrated high frequency of SSF consumption by coastal communities in Tuvalu and Kiribati (5.6 and 6.1 times per week, respectively)^22,23^. In these types of diets, nutrients from SSF, such as DHA + EPA fatty acids and vitamin B_12_, are especially important for public health. Importantly, intake of DHA + EPA is associated with reduced risk of heart disease^9^, and intake of vitamin B12 is associated with reduced risk of heart disease and cognitive decline^24^. Here, we show that small-scale fisheries have particularly high contribution to the supply of vitamin B12, calcium and DHA + EPA, although it can also be important for other nutrients depending on the nutritional value of the species consumed and availability of other foods.

There are many reasons to believe our findings are a conservative estimate of the contribution of small-scale fisheries to human nutrition. First, we assumed that coastal populations have the same national average intake of other food types (including other animal-sourced foods) based on the GND database. However, in coastal communities, fish can often be the only source of animal-sourced foods, and other food types are not accessible. Second, to calculate per capita consumption, we assumed that the entire coastal population within each country is consuming the small-scale fisheries catch. However, a large proportion of the catch can be sold in local markets around fishing communities. Many people living in larger cities along the coast might not consume small-scale fisheries catch and coastal communities might have higher than average seafood consumption. Therefore, average values presented here does not capture temporal and spatial variability in small-scale fisheries catch consumption across coastal populations. Third, even though the Sea Around Us database accounts for unreported catch, a portion of the small-scale fisheries production could still be unreported^17^. Fisheries that are destined for local consumption are particularly difficult to obtain production information and therefore can have higher levels of unreported catch^12^. Fourth, because of data limitations, we considered consumption of muscle tissue only (excluding bones, skin, etc.), which can be commonly consumed in some cultures. Lastly, we conservatively considered that artisanal catch is part of the international seafood trade. However, in many countries the artisanal catch is destined to the local markets, with only very few highly valuable species being exported^25^. Better data on these topics will increase the accuracy of the results presented here.

It is critical to understand whether SSF fisheries production is stable over time or potentially declining due to overfishing, climate change, pollution, habitat degradation and loss, or other threats that could have negative impacts on human nutrition. Nutritionally vulnerable coastal populations that depend on SSF and are particularly susceptible to fluctuations in catch and can benefit from sustained or increased catch through better resource management. Successful examples of small-scale fisheries governance demonstrate the value of securing fishing rights and empowering local communities^26–28^. Strategies that grant fishing rights to local communities (such as sustainable-use Marine Protected Areas, Territorial Use Rights for Fisheries, Locally Managed Marine Areas) incentivize management and promote sustainable practices^29–32^, and thus can promote human health through nutrition. The Voluntary Guidelines for Securing Sustainable Small-scale Fisheries developed by the Food and Agriculture Organization of the United Nations^10^ recognizes the importance of securing tenure rights to achieve fisheries sustainability. Such principles are drawn from practical experiences of small-scale fisheries governance from around the world and can be adopted by nutritionally sensitive countries that depend on small-scale fisheries for nutrition.

In addition to improved management, policies supporting access to nutrient-rich fish could have a positive impact on diets where undernutrition is prevalent. Aquatic foods are one of the most widely traded food products in the world, moving nutritious food from nutritionally vulnerable to nutrient-secure nations^33^. Although most SSF catch is consumed locally^16^, policies prioritizing local consumption and targeted to specific nutritional needs can help address inadequate nutrient intake and improve food security. Infrastructure and technological improvements that reduce food waste, improve food quality and safety, and help cope with inter-annual variation or declines can also play an important role in improving human health in regions where nutrients are most needed. This is particularly true in countries with nutritionally vulnerable populations with high dependence on small-scale fisheries.

## Materials and methods

### Predicting national seafood consumption by sector

We used the Sea Around Us (SAU)^34^ database for the year 2017 to assign seafood production to the different fisheries sectors. We used the fisheries sector categories from the database to divide the catch into commercial (artisanal and industrial) and non-commercial (recreational and subsistence). Artisanal fisheries are defined as small-scale and commercial; industrial fisheries defined as large-scale and commercial; recreational defined as fisheries for pleasure and non-commercial; and subsistence defined as small-scale and non-commercial^12^. We then removed the commercial catch that is discarded and destined to produce fish meal and fish oil, keeping only the production destined for direct human consumption (based on SAU database). Next, we added marine aquaculture production (hereafter referred as mariculture) from FAO to the total commercial production^18^. From the estimated total commercial production in each country, we then removed exports to estimate the total seafood that is consumed within each country (or apparent consumption). We used the FAO food balance sheets (FBS) and fish commodity categories (Table S1) to remove marine exports from the commercial production (artisanal, industrial and mariculture). For this, we assigned a fish commodity category to all species within total commercial production (based on SAU functional group), import and export statistics (Table S2). Next, we subtracted exports from commercial production based on the production proportion of each species and sector. For a few cases where more seafood was exported than was locally produced, negative production values within a species were set to zero. We subtracted the estimated percentage of freshwater crustaceans from exports, since our analysis focused only on marine species (Figure S10). Because countries might act as processing hubs^35^, we adjusted imports and exports to account for re-exports (imported seafood that is subsequently exported). Re-exports were estimated using two methods. First, we estimated total re-exports in 2017 using FAO trade data (Figure S11), which accounts for all seafood that is imported and re-exported without having undergone any type of transformation. Second, we estimated processed re-exports (imported seafood that is processed and exported) based on the difference between production and exports in the food balance sheets (Figure S12). Both estimates of re-exports were subtracted from exports and imports to avoid bias in seafood consumption estimates. Next, we subtracted estimated re-exports from imports to estimate the total imported seafood that is consumed in each country. Finally, we added all non-commercial production (recreational and subsistence derived from the SAU database) to the national seafood supply, assuming that the entire catch is destined for domestic consumption (see Figure S1 for a detailed flow chart).

### Predicting national seafood nutrient supply

To estimate nutrient supply from seafood production, we first multiplied the production of each species and sector by the estimated edible portion, according to the Aquatic Food Composition Database (AFCD)^4^. This step is important, because several parts of the seafood can be discarded and not consumed (e.g., bones, head, tail). Next, we assigned the nutritional content of each species using AFCD raw muscle tissue values. To assign a nutritional value for all nutrients and species, we used a hierarchical approach, giving sequential priority to (1) mean of species’ scientific name, (2) mean of species’ genus, (3) mean of species’ family, (4) mean of species’ order, (5) mean of species’ class, (6) mean of FAO fish commodities categories^4^. Through this approach, we were able to assign nutritional values for all species in the database, with about 70% of the species with nutrient value assigned on the family, genus or species level match on AFCD (see figure S13). Because imports in the food balance sheets are calculated only in the resolution of specific commodity groups, we assign a nutritional value based on the average nutrient content of each group (Table S1).

### Assigning coastal population nutrient supply

To predict the nutrient supply per capita, we assumed that catches from small-scale fisheries (artisanal and subsistence sectors) are either exported or consumed by the coastal population of the country (calculated from Grided Population of the World^36^). We acknowledge that in certain countries production from the artisanal sector can be distributed further inland, especially the most valuable species. However, there is widespread evidence that most of the catch from the artisanal sector is consumed in coastal cities and communities^37,38^. Coastal population size within each country was calculated by intersecting a buffer around coastlines with the raster of the gridded population of the world in 2019 ^36^. We calculated the population within 5, 10, 20, 30 and 40 and 50 km of the coastline (see figure S9 for sensitivity analysis). We used the sf package^39^ in R statistical software^40^ to perform all spatial analysis.

### Estimating the contribution of small-scale fisheries to nutrient supply

To calculate the importance of small-scale fisheries consumption to overall nutrient supply, we first estimated how much coastal communities are eating of all other food types (non-seafood). To this end, we conservatively assumed that the entire population in each country is consuming the national average of all other foods from the Global Nutrient Database (GND)^19^. The GND database takes the total estimated apparent consumption of all foods and calculates the total nutrient supply in each country. Apparent consumption is the estimated food consumed within each country, calculated as the production plus imports minus exports and food waste. We then used this database to estimate the total per capita nutrient supply of all non-seafood foods. With the estimated nutrient supply from all other sources (including the other seafood sectors), we then calculated the relative importance of small-scale fisheries to overall nutrient supply. Because seafood is the predominant source of DHA + EPA^8^, we assume that all the DHA + EPA is sourced from seafood producing sectors.

### Categorizing countries

Next, we categorized each country based on nutritional vulnerability and dependence on small-scale fisheries. We defined vulnerability to nutrient deficiencies according to previously published estimates of prevalence of inadequate intake^4^ across six key micronutrients (iron, zinc, calcium, DHA + EPA and vitamins A and B_12_). Vulnerability is assessed based on risk of undernourishment from available foods and does not consider the prevalence of diet-related metabolic disease or other health conditions. We considered a country reliant on SSF when overall dietary nutrient supply by SSF is above the 70th percentile across all assessed countries. We assumed this threshold based on estimates of inadequate intake, highlighting countries with inadequate intake greater than 50% (see sensitivity analysis for other threshold value assumptions). We combined these results with information on nutritional inadequacies of coastal countries^4^ to determine where nutrients from fish are most important.

### Sensitivity analysis

To assess the influence of our assumptions on the results, we performed sensitivity analyses for the main parameters of the analysis. For example, we tested how the results would change if we considered that the artisanal catch is entirely retained domestically versus exported into the international seafood market (Figure S4). In addition, we tested how the results are affected by the geographical distance criteria used to define the coastal population of a country, comparing results for populations within 10, 20, 30, 40 or 50 km from the coastline (Figure S8). Finally, we explored how the categorization of countries as reliant on SSF or nutritionally vulnerable is dependent on the assumed threshold value (70th percentile) (Figure S9).

## Supplementary Information

Supplementary Information.

## Data Availability

Data needed to evaluate the conclusions in the manuscript are available online or by request. Reconstructed catch data can be accessed through the Sea Around Us website (https://www.seaaroundus.org/data/#). FAO Fisheries and Aquaculture statistics can be accessed through the FishStatJ software (https://www.fao.org/fishery/en/statistics/software/fishstatj). Aquatic Foods Composition Database can be accessed through dataverse (https://dataverse.harvard.edu/dataverse/afcd). Additional data related to this paper may be requested from the authors. Codes used for analysis and visualization are available on GitHub (https://github.com/danielfvi).
