# Supplementary material for: Nutrient supply from marine small-scale fisheries
Source: Sci Rep. 2023 Jul 13;13:11357. doi: 10.1038/s41598-023-37338-z (PMC10344920; doi:10.1038/s41598-023-37338-z)
Supplement: Supplementary file 1 — Supplementary Information. [file 41598_2023_37338_MOESM1_ESM.pdf]

Supplementary information

Nutrient supply from small-scale fisheries

Viana et al.

Corresponding author: Daniel F. Viana (dviana@hsph.harvard.edu)

Supplementary figures

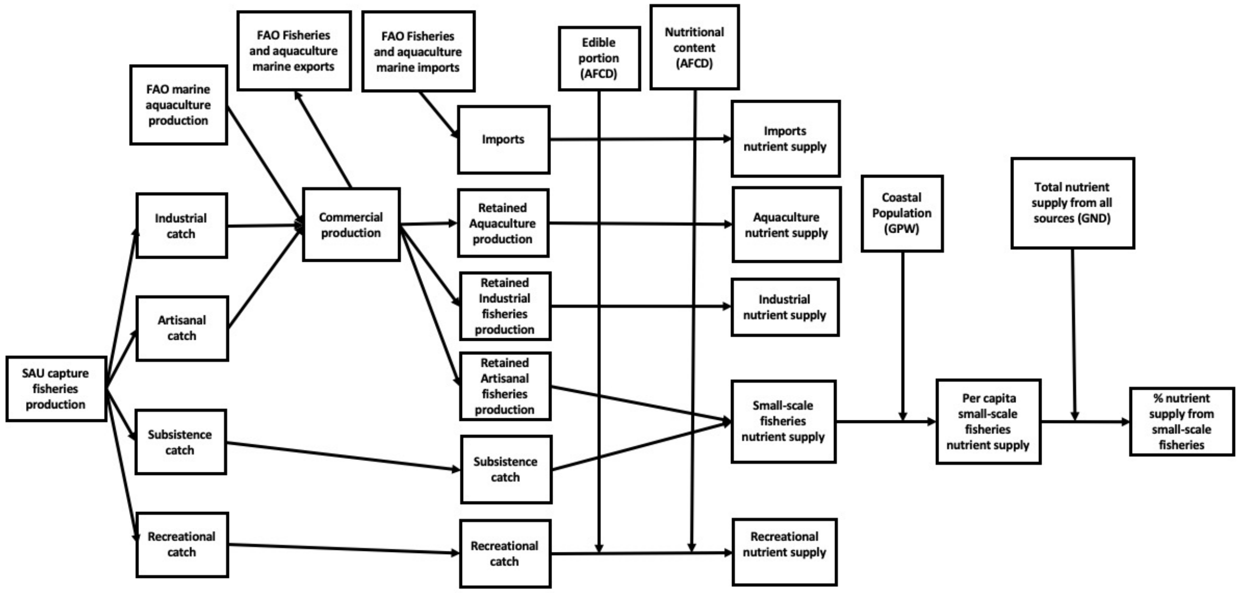

Figure S1. Model framework used to estimate the contribution of seafood producing sectors to human health and nutrition.

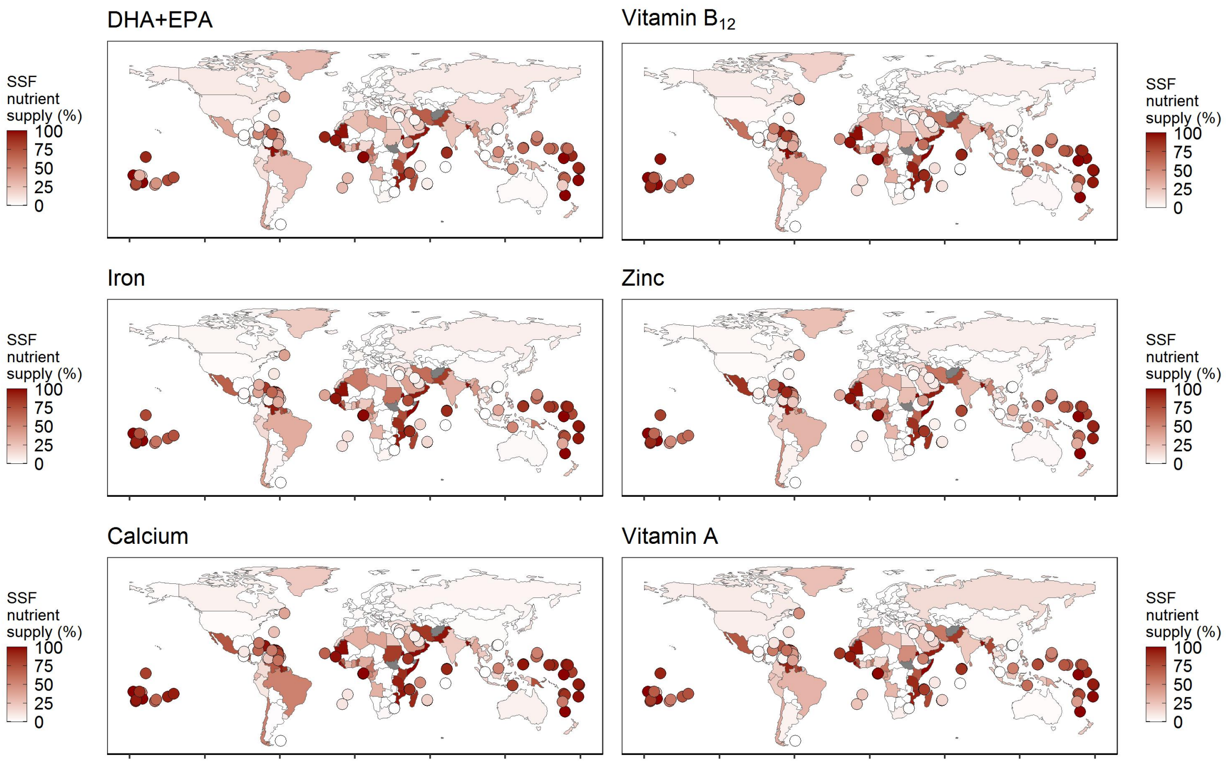

Figure S2 - Contribution of small-scale fisheries (SSF) to nutrient supply relative to total nutrient supply from all other seafood producing sectors (industrial and recreational fisheries, aquaculture and imports). Countries smaller than 25,000 km2 are illustrated as points.

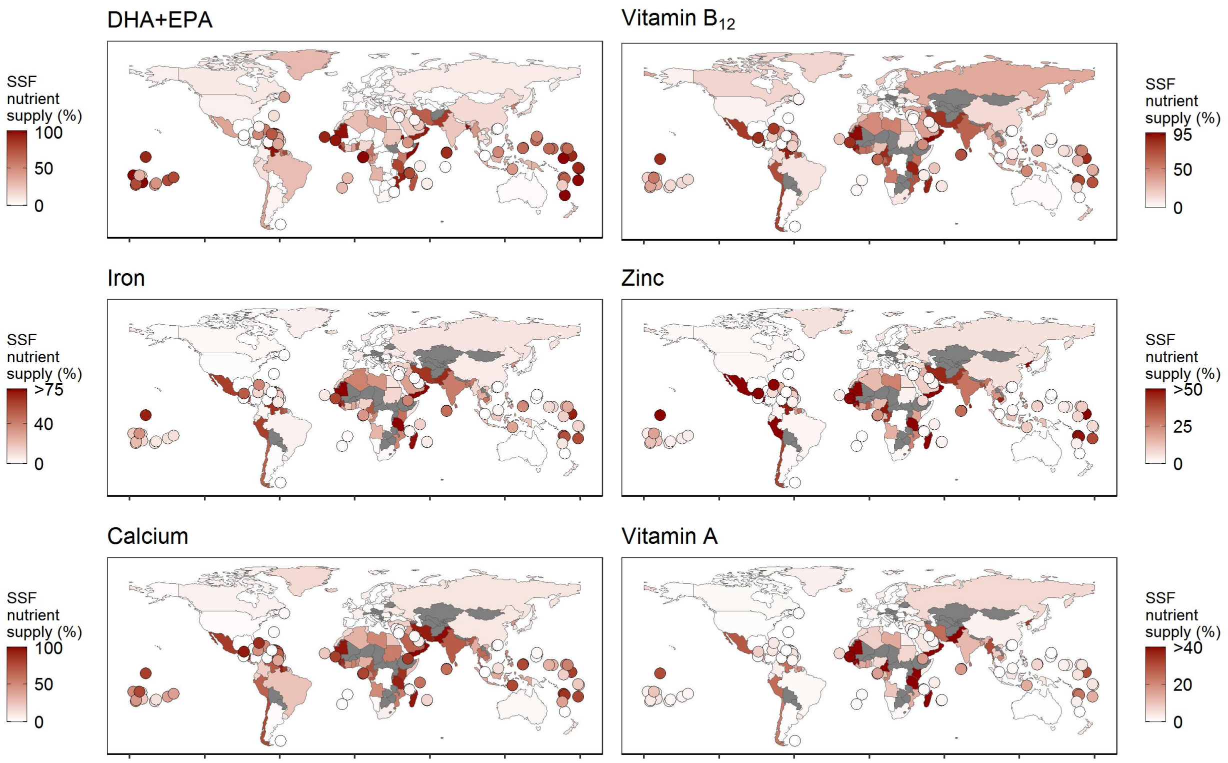

Figure S3 - Contribution of small-scale fisheries (SSF) to nutrient supply relative to total nutrient supply from all animal sourced foods (fish, beef, veal, pork, poultry and sheep). Countries smaller than 25,000 km2 are illustrated as points.

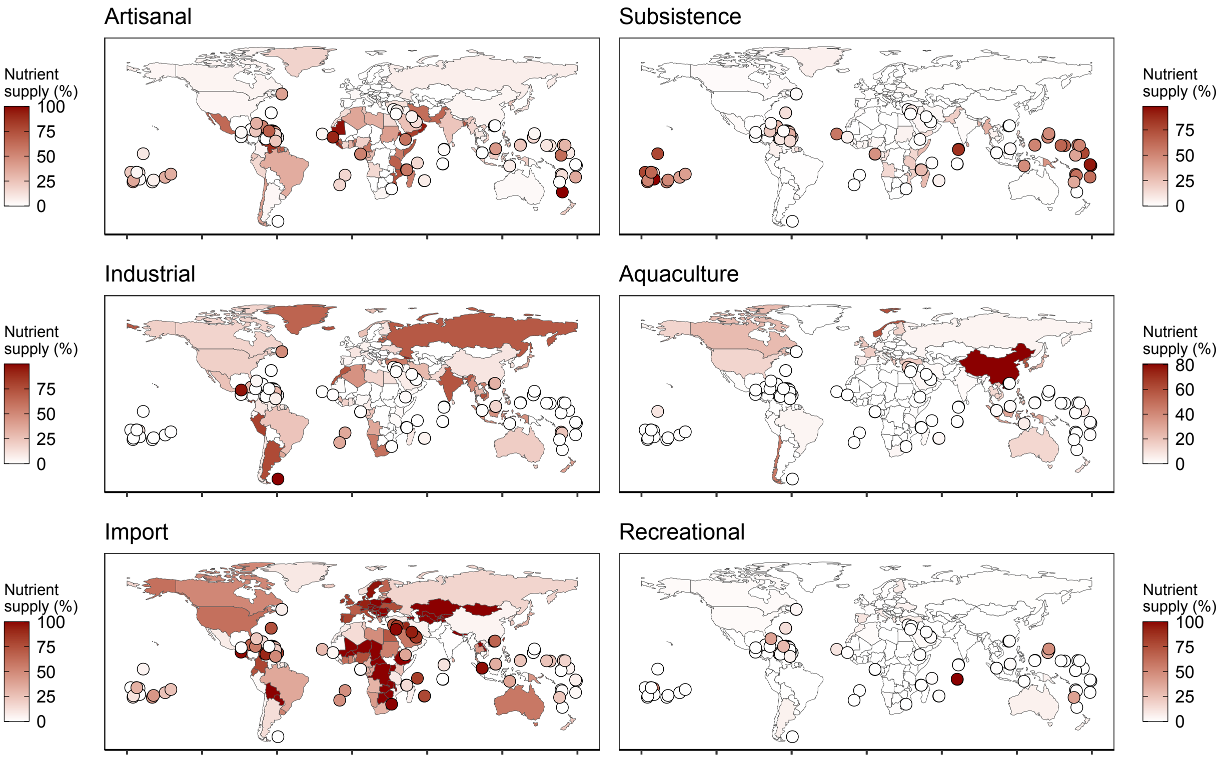

Figure S4 - Contribution of seafood sectors to nutrient supply relative to the total average nutrient supply from all seafood producing sectors. Nutrient supply is represented as the mean supply across iron, calcium, zinc, DHA+EPA, and vitamins A and B_{12}. Countries smaller than 25,000 km^2 are illustrated as points.

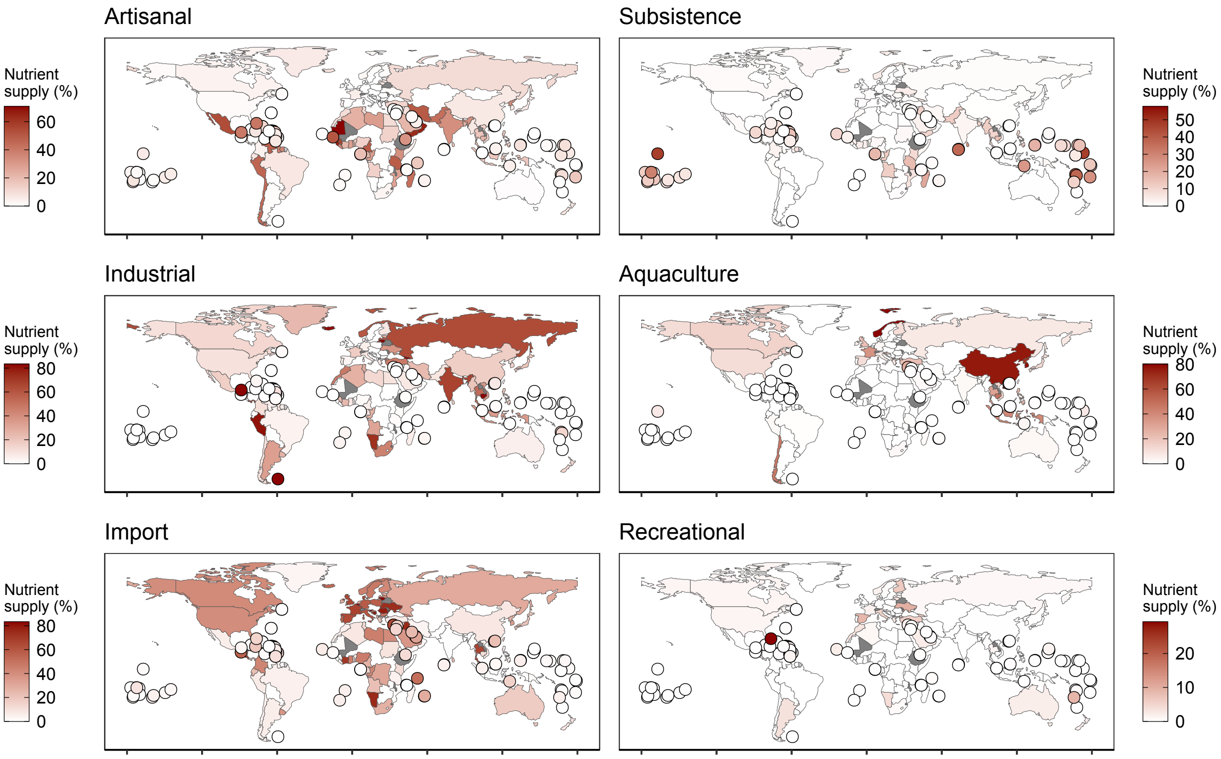

Figure S5 - Contribution of seafood sectors to nutrient supply relative to the average nutrient supply from all animal-sourced foods (fish, beef, veal, dairy, pork, poultry and sheep). Nutrient supply is represented as the mean supply across iron, calcium, zinc, DHA+EPA, and vitamins A and B_{12}. Countries smaller than 25,000 km^2 are illustrated as points.

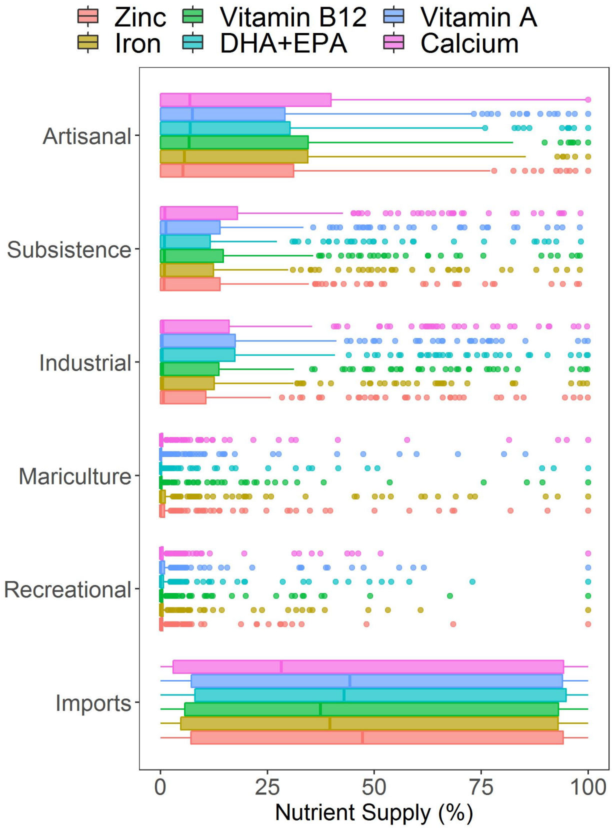

Figure S6 - Contribution of all seafood producing sectors to nutrient supply relative to total nutrient supply from all seafood producing sectors (additionally including industrial and recreational fisheries, and mariculture), where each point represents a country.

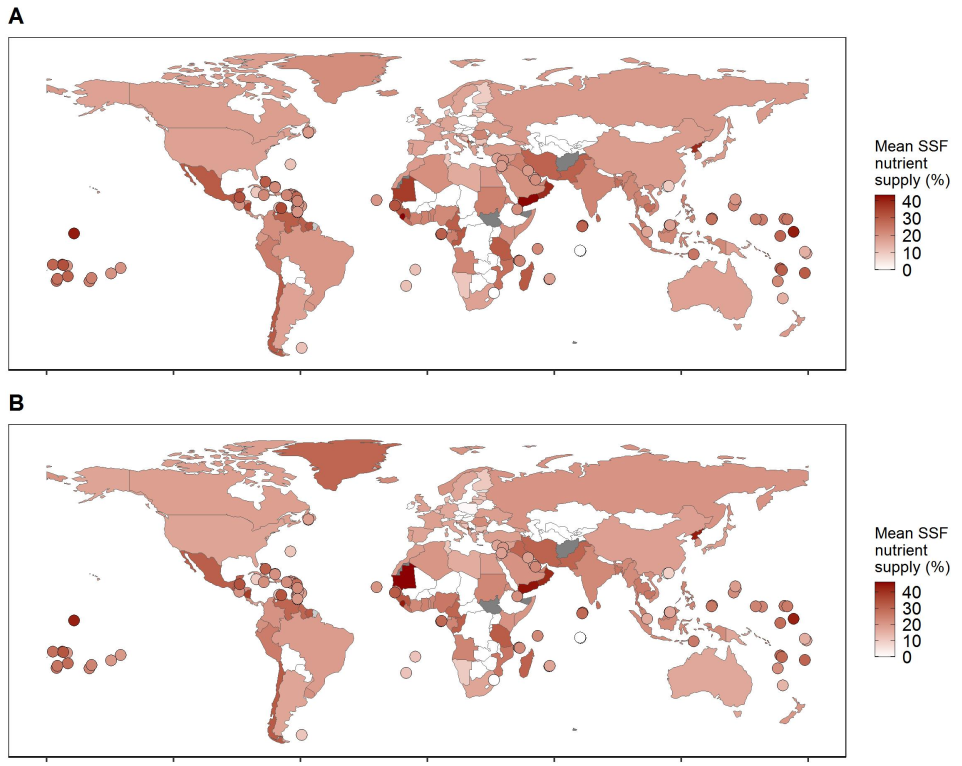

Figure S7 – Sensitivity analysis of the percentage of artisanal catch that is consumed within the country, where (A) represents a scenario where 100% of artisanal catch is part of the global seafood trade, thus catch is adjusted for trade and (B) represents a scenario where all production from artisanal catch is retained within the country thus non of the artisanal catch is exported. Figures represent the mean contribution of small-scale fisheries (SSF) to overall nutrient supply across nutrients that are abundant in aquatic species and are important for human health (iron, calcium, zinc, protein, vitamin B12 and DHA+EPA).

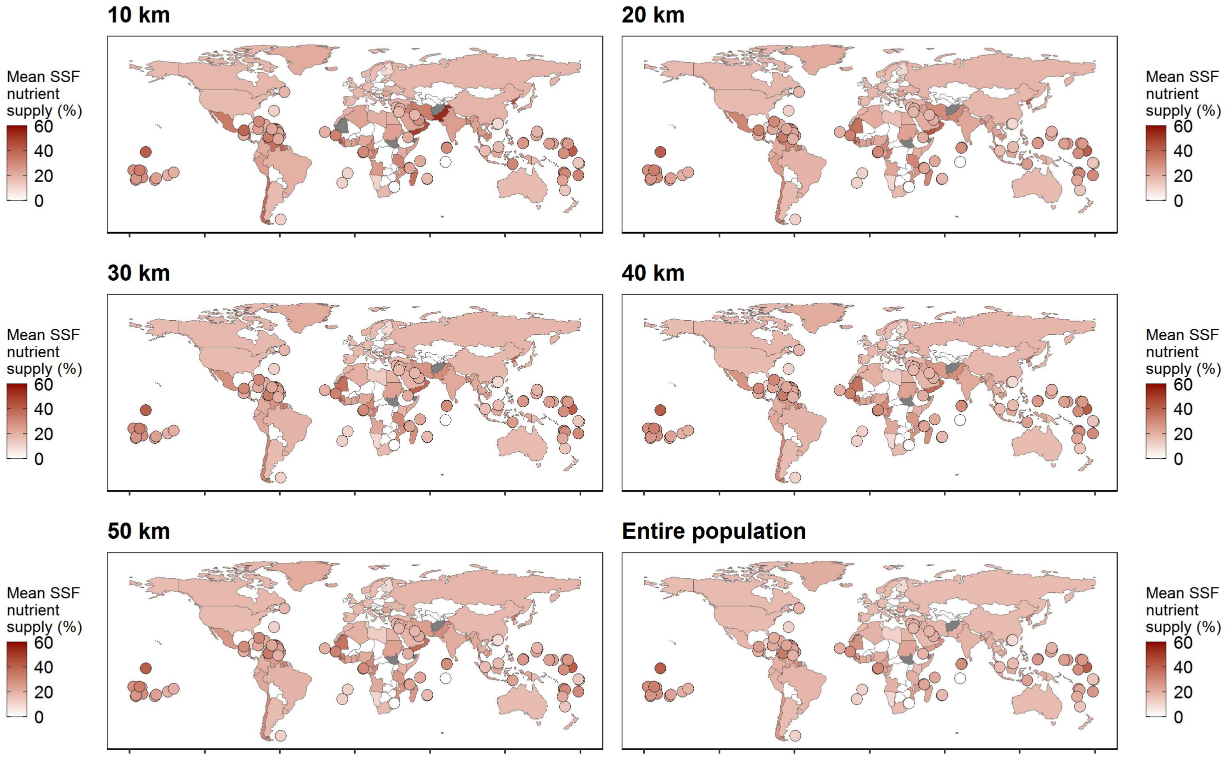

Figure S8 – Sensitivity analysis of the threshold for calculating coastal population within each country. Figures represent the mean contribution of small-scale fisheries (SSF) to overall nutrient supply across nutrients that are abundant in aquatic species and are important for human health (iron, calcium, zinc, protein, vitamin B12 and DHA+EPA).

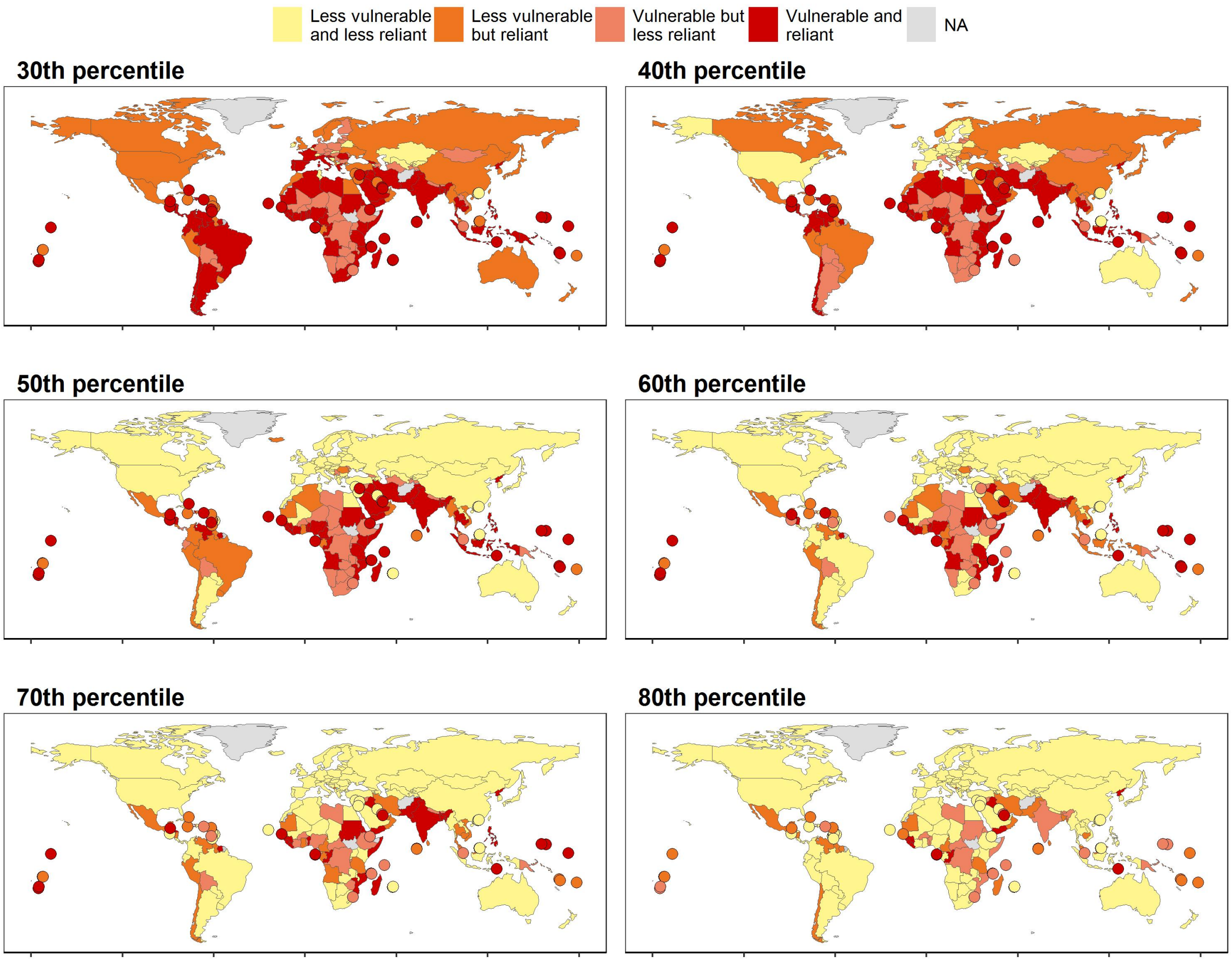

Figure S9 - Sensitivity analysis of the assumed threshold for considering a country reliant on small-scale fisheries and nutritionally vulnerable. Figures categorize countries that have high prevalence of inadequate micronutrient intake (vulnerable) and that rely on SSF for the supply of key nutrients (reliant) across nutrients that are abundant in aquatic species and are important for human health (iron, calcium, zinc, protein, vitamin B12 and DHA+EPA).

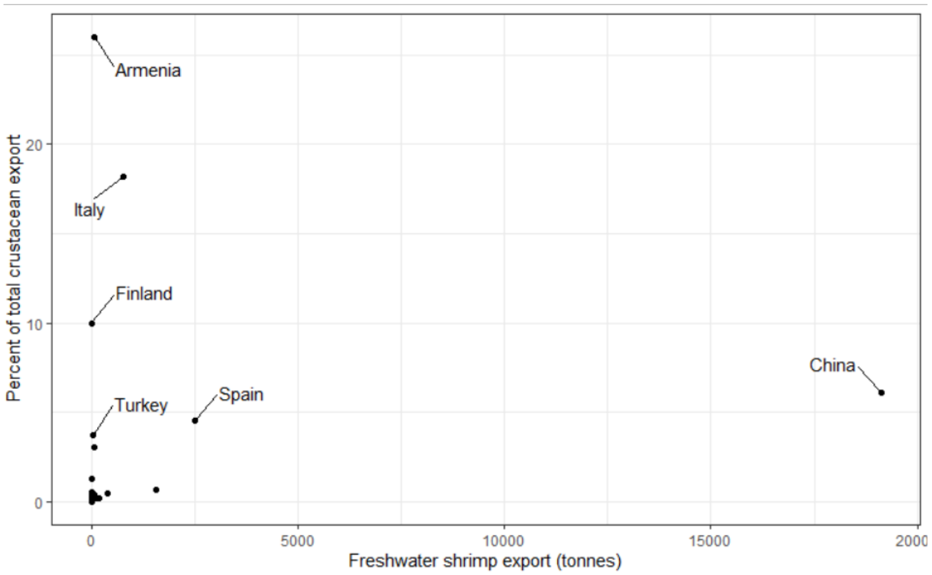

Figure S10 - Estimated percentage of freshwater crustaceans exports based on FAO trade data.

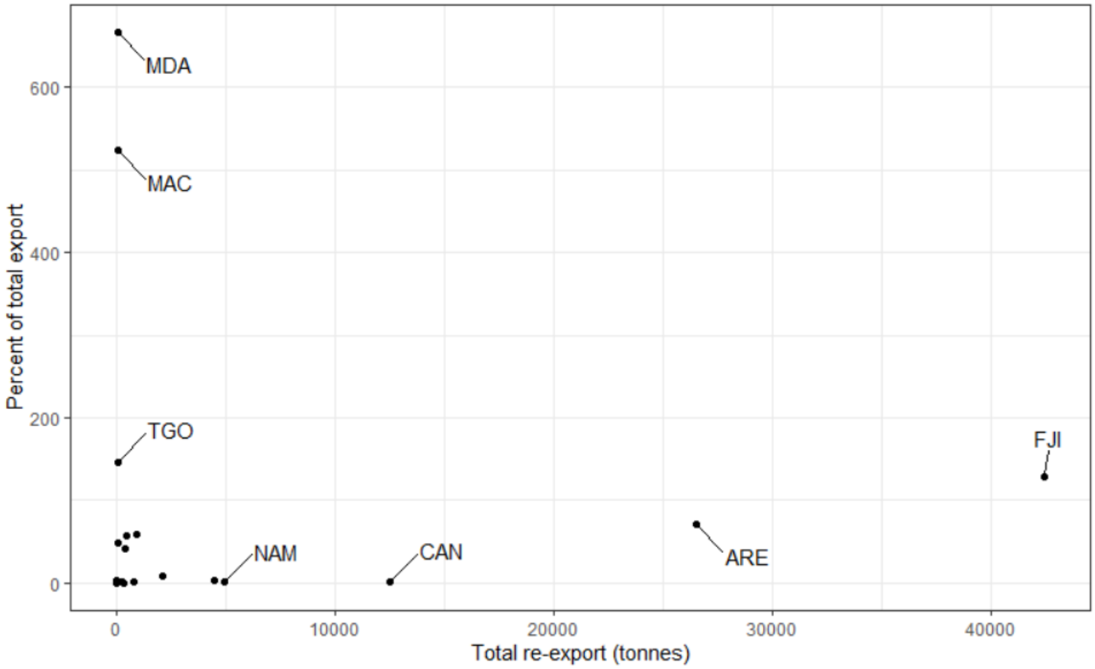

Figure S11 - Estimated percentage of re-exports from total exports based on FAO trade data.

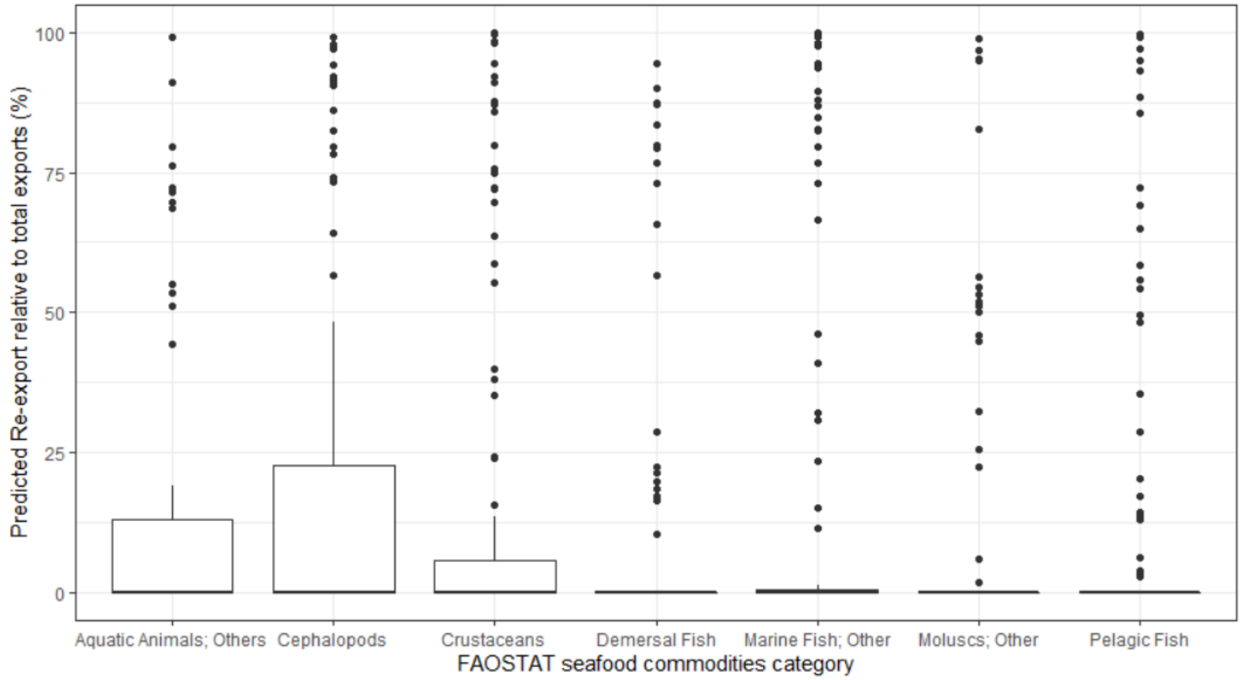

Figure S12 - Estimated percentage of re-exports based on the difference between production and exports in FAO's food balance sheets.

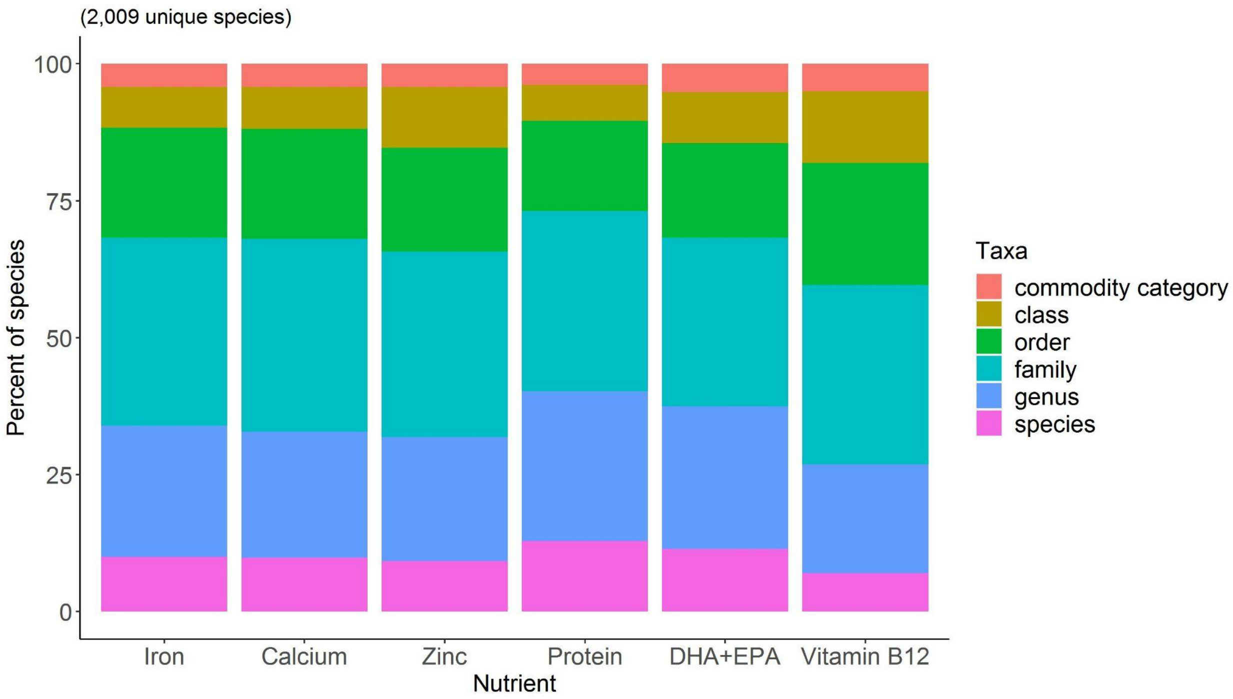

Figure S13 - Total number of species per nutrient and criteria used to fill nutritional values from the Aquatic Foods Composition Database (AFCD). For all nutrients, there are a total of 2,143 unique species derived from disaggregation efforts.

Supplementary tables

Table S1. FAO commodities categories used to assign exports to seafood production and their respective average nutrient content.

**Category**	**Nutrient**						
	Calcium (mg/100g)	Iron (mg/100g)	Omega-3 fatty acids (g/100g)	Protein (g/100g)	Vitamin A (mg/100g)	Vitamin B12 (ug/100g)	Zinc (mg/100g)
Aquatic Animals; Others	539.13	4.44	0.22	12.91	1.26	2.04	1.61
Cephalopods	38.35	3.03	0.30	17.27	3.83	3.34	1.67
Crustaceans	270.84	2.61	0.49	17.54	1.16	3.36	2.13
Demersal Fish	105.84	1.32	0.75	18.42	4.24	1.91	0.88
Freshwater Fish	165.72	1.56	1.22	18.60	2.96	3.61	1.13
Marine Fish; Other	129.46	1.60	0.92	19.31	3.86	3.58	1.00
Moluscs; Other	142.61	5.19	0.30	14.56	2.94	6.58	1.79
Pelagic Fish	153.08	1.88	1.08	20.20	3.49	5.25	1.13

Table S2. Matching of functional groups within the Sea Around Us (SAU) database to FAO group of commodities.

**Functional Group**	**Commodity category**
Jellyfish	Aquatic Animals; Others
Cephalopods	Cephalopods
Shrimps	Crustaceans
Lobsters, crabs	Crustaceans
Krill	Crustaceans
Large sharks (>=90 cm)	Demersal Fish
Large rays (>=90 cm)	Demersal Fish
Small to medium flatfishes (<90 cm)	Demersal Fish
Large demersals (>=90 cm)	Demersal Fish
Small reef assoc. fish (<30 cm)	Demersal Fish
Large reef assoc. fish (>=90 cm)	Demersal Fish
Medium reef assoc. fish (30 - 89 cm)	Demersal Fish
Small bathydemersals (<30 cm)	Demersal Fish

Large bathydemersals (>=90 cm)	Demersal Fish
Small demersals (<30 cm)	Demersal Fish
Large flatfishes (>=90 cm)	Demersal Fish
Small to medium rays (<90 cm)	Demersal Fish
Medium bathydemersals (30 - 89 cm)	Demersal Fish
Small to medium sharks (<90 cm)	Demersal Fish
Medium demersals (30 - 89 cm)	Demersal Fish
Other demersal invertebrates	Moluscs; Other
Medium pelagics (30 - 89 cm)	Pelagic Fish
Small pelagics (<30 cm)	Pelagic Fish
Large benthopelagics (>=90 cm)	Pelagic Fish
Small benthopelagics (<30 cm)	Pelagic Fish
Medium benthopelagics (30 - 89 cm)	Pelagic Fish
Large pelagics (>=90 cm)	Pelagic Fish
Large bathypelagics (>=90 cm)	Pelagic Fish
Medium bathypelagics (30 - 89 cm)	Pelagic Fish

Small bathypelagics (<30 cm)	Pelagic Fish
